# Entomopathogenic fungi *Beauveria bassiana* and *Metarhizium anisopliae* play roles of maize (*Zea mays*) growth promoter

**DOI:** 10.1038/s41598-022-19899-7

**Published:** 2022-09-20

**Authors:** Yinmei Liu, Youkun Yang, Bin Wang

**Affiliations:** Provincial Key Laboratory of Microbial Control, Anhui Agricultural University, Hefei, 230036 China

**Keywords:** Developmental biology, Microbiology, Physiology, Plant sciences

## Abstract

*Beauveria bassiana* and *Metarhizium anisopliae* are two of the most important and widely used entomopathogenic fungi (EPFs) to control insect pests. Recent studies have revealed their function in promoting plant growth after artificial inoculation. To better assess fungal colonization and growth-promoting effects of *B. bassiana* and *M. anisopliae* on crops, maize *Zea mays* seedlings were treated separately with 13 *B. bassiana* and 73 *M. anisopliae* as rhizosphere fungi in a hydroponic cultural system. Plant growth indexes, including plant height, root length, fresh weight, etc., were traced recorded for 35 days to prove the growth promoting efficiency of the EPFs inoculation. Fungal recovery rate (FRR) verified that both *B. bassiana* and *M. anisopliae* could endophytically colonize in maize tissues. The recovery rates of *B. bassiana* in stems and leaves were 100% on the 7th day, but dropped to 11.1% in the stems and 22.2% in the leaves on the 28th day. Meanwhile, *B. bassiana* was not detected in the roots until the 28th day, reaching a recovery rate of 33.3%. *M. anisopliae* strains were isolated from the plant roots, stems and leaves throughout the tracing period with high recovery rates. The systematical colonization of *B. bassiana* and *M. anisopliae* in different tissues were further corroborated by PCR amplification of fungus-specified DNA band, which showed a higher detection sensitivity of 100% positive reaction. Fungal density comparing to the initial value in the hydroponic solution, dropped to be well below 1% on the 21st day. Thus, the two selected entomopathogenic fungal strains successfully established endophytic colonization rather than rhizospheric colonization in maize, and significantly promoted its growth in a hydroponic cultural system. Entomopathogenic fungi have great application potential in eco-agricultural fields including biopesticides and biofertilizers.

## Introduction

Entomopathogenic fungi (EPFs) have been proven as important biological control agents (BCAs) against various pests due to their wide host range, simple production, good durability and strong virulence^1–3^. *Beauveria bassiana* and *Metarhizium anisopliae*, were commercially used as a sustainable control strategy for key insect pests of maize, including corn borer *Ostrinia furnacalis and Helicoverpa armigera* in China, to avoid the abuse of chemical pesticides^4^*.* In fungi-based pest management, the triangle relationship among plants, insect pests and the fungi is of much higher complexity over the relationship between insect pests and fungal pathogens.

Many plants are symbiotic with endophytic fungi^5^, which live in plant tissues and do no obvious harm to plants^6^. Endophytic fungi are inherent organisms after establishing a mutually beneficial and cooperative symbiosis with their hosts^7^, and they can directly or indirectly promote plant growth and improve plant adaptability to adversity, including biological and abiotic stress^8–10^. Endophytes have important phylogenetic and lifestyle diversity characteristics, such as colonization, transmission, plant host specificity and colonization in different plant tissues^11^. Employing EPFs as endophytic fungi has aroused increasing interest from researchers and has unfolded many unique benefits as compared to their traditional counterparts.

*B. bassiana* and *M. anisopliae* can colonize a variety of plants, including but not limited to wheat, soybean, rice, bean, onion, tomato, palm, grape, potato and cotton^12^. Local or systematic colonization can happen mainly in the roots, stems, leaves and internal tissues of plants^11^. The fungal endophytic colonization can promote plant growth after artificial inoculation with seed treatment, foliar spraying and soil irrigation, etc.^13–16^. Treating crop seed with *B. bassiana* and *M. anisopliae* resulted in successful endophytic colonization in crop tissues and promoted crop growth, as evidenced by stem height, root length, fresh root and fresh stem weight^17–19^. Soil inoculation and foliar spraying of *B. bassiana* are also the most used application method which considerably enhanced the growth of corn seedlings^20^.

The purpose of this study was to evaluate growth-promoting effects and colonization behavior of *B. bassiana* and *M. anisopliae* on maize (*Zea mays*) seedlings and the influence on the plant growth under a hydroponic cultural system.

## Results

### Effects of *B. bassiana* and *M. anisopliae* on the growth of maize seedlings

**Figure 1:**
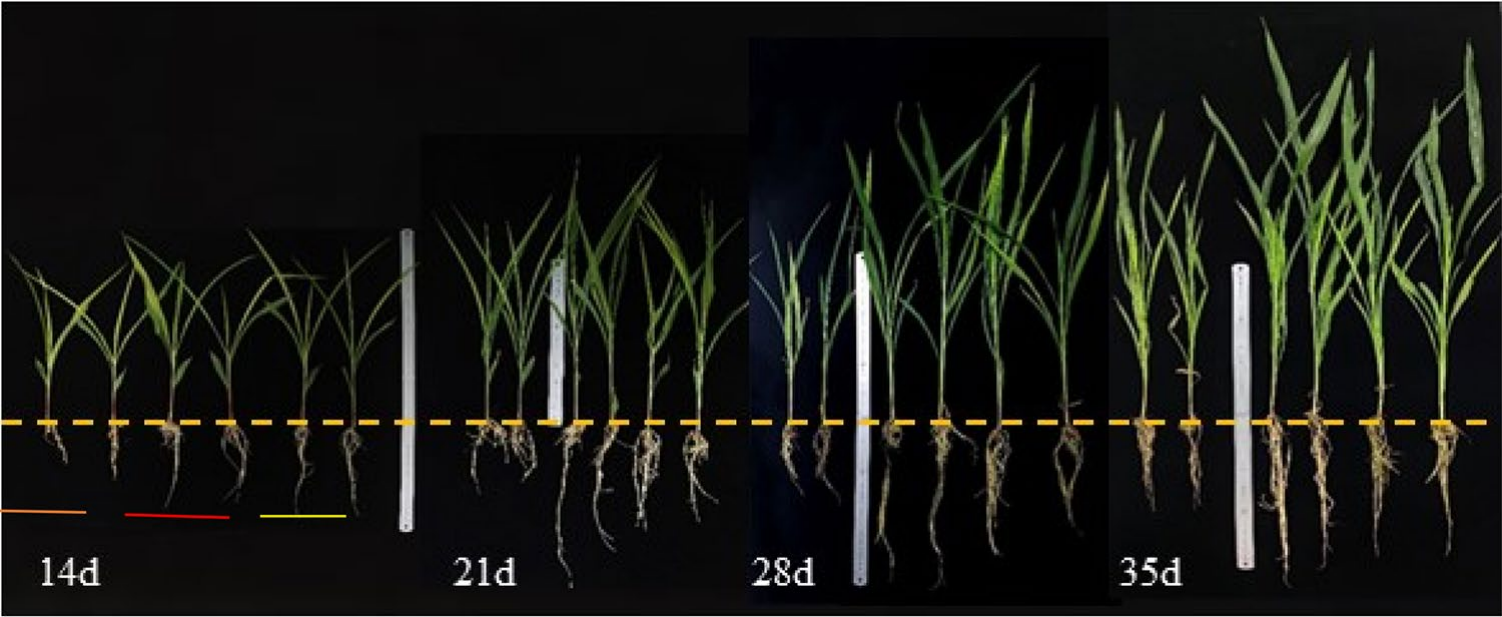
Growth status of maize seedlings in different treatments over time. From left to right different colour line represents maize seedlings of Control, *B. bassiana* treatment and *M. anisopliae* treatment, respectively.

Both *B. bassiana* and *M. anisopliae* treatment led to an apparently positive effects on maize growth as observed during the 35-day period. As shown in Fig. 1, the growth promotion effect of the fungi on different maize organs is dependent on the growing stages.

**Figure 2:**
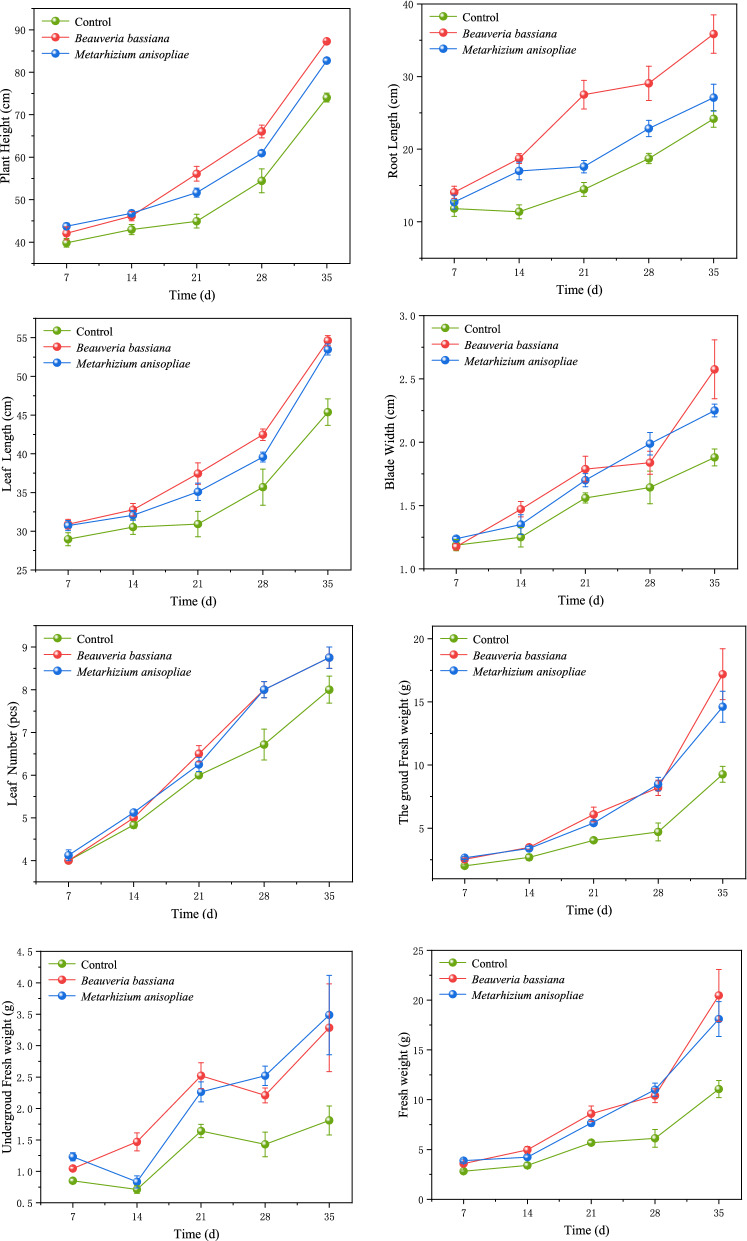
Temporal effects of *B. bassiana* and *M. anisopliae* on growth indexes of maize.

On the 7th day, the aboveground fresh weight (df = 23, F = 12.647, P = 0.000), underground fresh weight (df = 23, F = 18.857, P = 0.000) and total fresh weight (df = 23, F = 18.762, P = 0.000) of maize seedlings in the treatment groups were significantly higher (> 20%) than those in the control group, while other indexes showed no observable difference (Table 1). On the 28th day, crop height (df = 23, F = 10.452, P = 0.001), root (df = 23, F = 10.109, P = 0.001), leaf length (df = 23, F = 6.092, P = 0.009) and the aboveground fresh weight (df = 23, F = 11.310, P = 0.001) values were significantly higher in the treatment groups (Figs. 1 and 2). On the 35th day (Table 2), plant height (P = 0.000), root length(P = 0.003), leaf length (P = 0.002), leaf width (P = 0.015), leaf number (P = 0.1118), above-ground fresh weight (P = 0.004), underground fresh weight (P = 0.062) and total fresh weight (P = 0.007) values of the *B. bassiana* treated group were 17.87%, 48.26%, 20.37%, 36.97%, 9.38%, 85.54%, 81.49% and 84.87% higher than those of the control groups, respectively. Meanwhile, as learned in the *M. anisopliae* treated group, plant height (P = 0.000), root length (P = 0.203), leaf length (P = 0.005), leaf width (P = 0.004), leaf number (P = 0.118), above-ground fresh weight(P = 0.004), underground fresh weight(P = 0.029) and total fresh weight (P = 0.006) were 11.76%,12.08%, 17.84%, 19.68%,9.38%,57.74%, 92.71% and 63.45% higher than those of the control group.

**Table 1:** Effects of *B. bassiana* and *M. anisopliae* on biomass of maize seedlings after 7 days of treatment.

Treatments	Control	*B. bassiana* treatment	*M. anisopliae*treatment	df	F	P-value
Plant height (cm)	39.838 ± 1.023a	42.075 ± 1.517a	43.738 ± 0.771a	23	2.913	0.076
Root length (cm)	11.825 ± 1.077a	14.063 ± 0.837a	12.713 ± 0.957a	23	1.32	0.275
Leaf length (cm)	28.963 ± 0.842a	30.900 ± 0.640a	30.725 ± 0.656a	23	2.225	0.133
Blade width (cm)	1.188 ± 0.044a	1.175 ± 0.025a	1.238 ± 0.018a	23	1.131	0.342
Leaf number (pcs)	4.000 ± 0.000a	4.000 ± 0.000a	4.125 ± 0.125a	23	1.000	0.385
Aboveground fresh weight (g)	2.018 ± 0.081b	2.536 ± 0.105a	2.651 ± 0.098a	23	12.647	0.000
Underground fresh weight (g)	0.805 ± 0.042b	1.046 ± 0.034a	1.231 ± 0.066a	23	18.857	0.000
Total fresh weight (g)	2.823 ± 0.114b	3.583 ± 0.113a	3.883 ± 0.147a	23	18.762	0.000

The same lowercase letters in the same column indicates means ± SE are not significantly different using Tukey HSD test at p = 0.05 level of significance; the same as below.

**Table 2:** Effects of *B. bassiana* and *M. anisopliae* on biomass of maize seedlings after 35 days of treatment.

Treatments	Control	*B. bassiana* treatment	*M. anisopliae* treatment	df	F	P-value
Plant height(cm)	74.020 ± 1.047c	87.250 ± 0.065a	82.725 ± 0.614b	23	77.543	0.000
Root length(cm)	24.180 ± 1.148b	35.850 ± 2.642a	27.100 ± 1.847b	23	10.482	0.004
Leaf length(cm)	45.380 ± 1.709b	54.625 ± 0.650a	53.475 ± 0.715a	23	7.079	0.012
Blade width(cm)	1.880 ± 0.066b	2.575 ± 0.232a	2.250 ± 0.050ab	23	17.770	0.001
Leaf number (pcs)	8.000 ± 0.316a	8.750 ± 0.250a	8.750 ± 0.250a	23	10.038	0.004
The ground fresh weight (g)	9.264 ± 0.625b	17.188 ± 2.004a	14.613 ± 1.228a	23	2.473	0.134
Underground fresh weight (g)	1.810 ± 0.231b	3.285 ± 0.697a	3.488 ± 0.631a	23	3.312	0.079
The total fresh weight (g)	11.074 ± 0.850b	20.473 ± 2.602a	18.100 ± 1.742a	23	8.282	0.008

### Population dynamics of *B. bassiana* and *M. anisopliae* in the hydroponic solution

The *B. bassiana* population decreased sharply from 5.92 × 10^3^ to 22.3 CFUs/ml over the first week and 7.20 CFUs/ml over the second week. *B. bassiana* wasn’t detected in the hydroponic solution on the 21st day (Table 3). The *M. anisopliae* population also exhibited a rapid decrease from the initial density of 8.56 × 10^3^ to 5.55 × 10^2^ CFUs/ml over the first week. Then, 1.27 × 10^2^ CFUs/ml and 17 CFUs/ml were detected on the 14th and 21st days respectively, while the solution was free of *M. anisopliae* on the 28th day.

**Table 3:** Population dynamic of *B. bassiana* and *M. anisopliae* in the hydroponic solution in terms of CFUs (cps/ml).

Treatment	Days				
	0	7	14	21	28
*B. bassiana* treated	(5.92 ± 0.26) × 10^3^	22.30 ± 10.33	7.20 ± 2.99	–	–
*M. anisopliae* treated	(8.56 ± 0.90) × 10^3^	(5.55 ± 0.22) × 10^2^	(1.27 ± 0.12) × 10^2^	(1.70 ± 0.51) × 10	–

### Fungal endophytic, temporal and spatial distribution in maize tissues

#### Isolation and identification of endophytic *B. bassiana* and *M. anisopliae* from different tissues of maize

**Figure 3:**
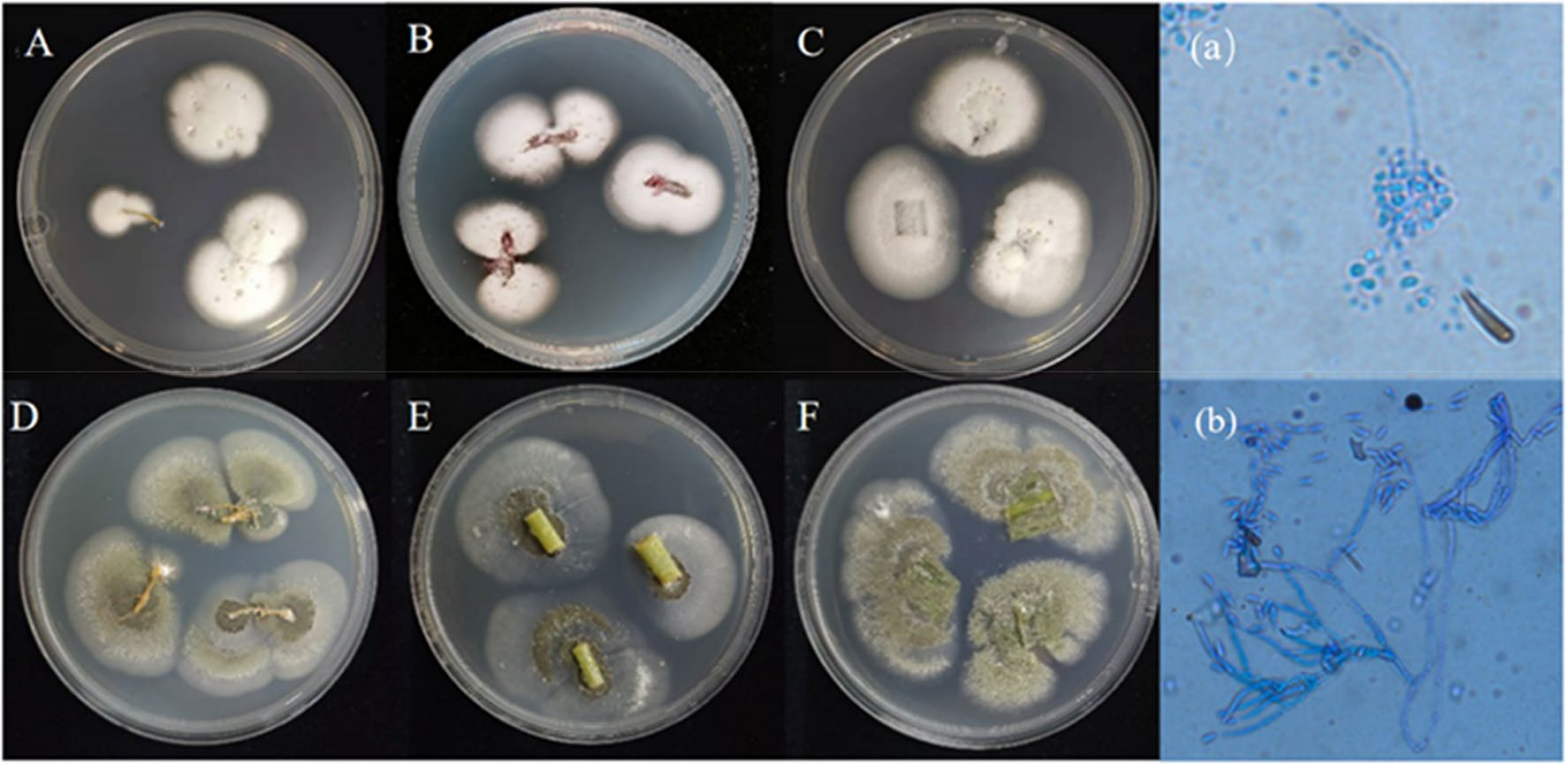
Fungal colonies of entomopathogenic fungi from maize seedling tissues on selective medium *B. bassiana* treatment group: A: Root; B: Stem; C: Leaf; (**a**) Morphology microscope of isolated fungi *M. anisopliae* treatment group: D: Root; E: Stem; F: Leaf; (**b**) Electron microscope of isolated fungi.

Fungal colonies began to appear 4 days after the cultivation. The spore morphology, mycelial characteristics and colony characteristics of the isolated strains were observed under light microscope. The fungal conidiogenous cell of *B. bassiana* formed a zigzag curve, and the conidia were spherical, transparent and smooth, consistent with the morphology of the pure strain *B. bassiana* Bb-13^21^ (Fig. 3a). The fungal colonies of *M. anisopliae* were white in the beginning and turned olive green after sporulation. The hyphae were transparent, separated and branched. Bottle-shaped conidiogenous cells formed a long string of conidia in basal continuity (Fig. 3b). The conidia were elliptical, consistent with the morphology of pure strain Ma73. In general, *B. bassiana* (Fig. 3A–C) and *M. anisopliae* (Fig. 3D–F) were isolated from maize tissues by culturing tissue samples on the fungal selective medium, verifying their endophytic colonization in maize root, stem and leaf.

**Figure 4:**
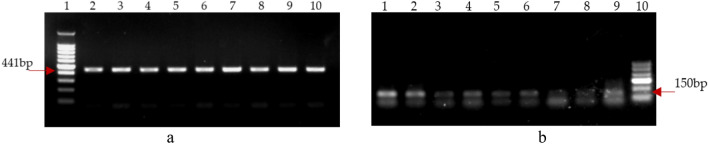
PCR amplification of genomic DNA extracted from different plant parts of *B. bassiana* and *M. anisopliae* treated *Zea mays* (**a**) Bb13; Lanes 1, 100 bp—1 kb DNA ladder; Lane 2–4, DNA from *Zea mays* root; Lane 5–7, DNA from *Zea mays* steam; Lane 8–10, DNA from *Zea mays* leaf. (**b**) Ma73; Lanes 10, 100—600 bp DNA ladder; Lane 1–3, DNA from *Zea mays* root; Lane 4–6, DNA from *Zea mays* steam; Lane 7–9, DNA from *Zea mays* leaf.

PCR amplification of special DNA band for *B. bassiana(*441 bp) and *M. anisopliae*(150 bp) from different tissues of fungal treated maize seedlings was also used to trace endophytic colonization of *B. bassiana* (Fig. 4a) and *M. anisopliae* (Fig. 4b) in crop tissues. This method was proved to be highly sensitive. Maize tissues, in which *B. bassiana* or *M. anisopliae* colony could not be detected on the selective medium, all responded to positive PCR amplification. Genomic DNA extracted from maize seedling tissues of the control group failed to amplify.

#### Temporal and spatial colonization of the two entomopathogenic fungi in maize tissues

As discussed above, both *B. bassiana* and *M. anisopliae* successfully colonized in different tissues of maize seedlings. The fungal recovery rate (FRR) of *B. bassiana* or *M. anisopliae* from different tissues of maize seedlings revealed fungal temporal and spatial colonization in maize tissues (Table 4). On the 7th day after fungal inoculation, 100% FRRs were observed in stems and leaves for *B. bassiana* and *M. anisopliae*, while FRR from the roots was only 11.1% for *M. anisopliae*, and 0% for *B. bassiana*. *M. anisopliae* was successfully detected in maize roots, leaves and stems in every sampling time with a fluctuating FRR, reaching almost 100% FRRs in all tissues. For *B. bassiana*, FRR was relatively lower than that of *M. anisopliae*. Only on the 28th day, *B. bassiana* was detected in all maize organs. Fungal endophytic behavior showed somewhat fungal species specificity.

**Table 4:** Recovery rate of *B. bassiana* and *M. anisopliae* from maize tissues.

Days	Recovery rate of *M. anisopliae*(%)			Recovery rate of *B. bassiana*(%)		
	The root	Stem	Leaf	The root	Stem	Leaf
7	11.1	100	100	–	100	100
14	22.2	66.7	33.3	–	–	–
21	88.9	11.1	100	–	22.2	22.2
28	55.6	11.1	55.6	33.3	11.1	22.2
35	100	88.9	100	–	–	–

The colonization of *B. bassiana* and *M. anisopliae* in maize tissues was further detected by PCR amplification. Table 5 showed that the colonization rates of *B. bassiana* in tissues of all maize organs were 100% in each sample time (7–35 days). The same results were observed in leaf tissues for *M. anisopliae*, but the fungus was not constantly detected at 100% in maize stems and leaves.

**Table 5:** Recovery rate of *B. bassiana* and *M. anisopliae f*rom maize tissues by PCR amplification.

Days	Recovery rate of *M. anisopliae* (%)			Recovery rate of *B. bassiana* (%)		
	The root	Stem	Leaf	The root	Stem	Leaf
7	100	100	100	100	100	100
14	–	100	100	100	100	100
21	100	100	100	100	100	100
28	33.3	66.7	100	100	100	100
35	66.7	66.7	100	100	100	100

## Discussion

When compared with traditional soil cultural systems, hydroponic culture systems are popular for industrial production of vegetables, but not so often for ecological study between microbes and plants. In this study, a hydroponic cultural system instead of a soil cultural system was used to assess fungal colonization and growth-promoting effects of *B. bassiana* and *M. anisopliae* on maize. Soil, as the most widely used culture substrate, might obscure the relationship between entomopathogenic fungi and maize crops, due to the complexities involved in a soil cultural system including soil microorganisms, soil organic matter, soil textural composition et al. In a hydroponic culture system, environmental factors are simple and constant, making repeatable results and clearer elucidation of the biological relationship just between fungi and plants possible. After inoculation, *B. bassiana* or *M. anisopliae* were isolated and PCR detected from the maize stems, leaves and roots, proving fungal transfer in maize tissues. These results were in consistent with the observation of Spiridon Mantzoukas et al.^2^, where fungal transfer was confirmed by isolating fungi from different parts of leaves (7 days, 14 days, 21 days and 35 days), roots (35 days), stems (35 days) and buds (35 days). Thus, fungal transfer in maize tissues could happen in the hydroponic culture system.

FRRs from different maize tissues showed temporal and spatial dynamics for *B. bassiana* and *M. anisopliae*. For both fungi, maize leaves were ideal sites over time, somewhat different from Akutse’s research, where fungus was hardly detected in bean leaf tissue^22^. As monitored by culturing maize tissues on the fungal selective medium, FRRs showed a dependence on the type of plant tissues. Certain endophytic fungi exhibit a degree of tissue specificity as they adapt to existing conditions in each organ^23–26^. Guo et al.^27^ reported that the tissue specificity of endophytic fungi results from their adaptation to the unique conditions of the plant organs.

Inoculation method is crucial to fungal colonization pattern^28^. Parsa et al.^29^ found that *B. bassiana* could colonize endogenously when the plants were treated by spraying or irrigating, while the roots could only be colonized only by irrigation. In sorghum, Tefera and Vidal reported higher rates of *B. bassiana* colonization in stems by leaf inoculation, and higher rates in roots and stems by seed inoculation. In the current work, root inoculation of both fungi was conducted by adding conidia suspensions directly into the hydroponic cultural system. This method might enhance the fungal transmission efficiency since the flowing water can help the fungal conidia to move toward maize crop roots. Beside inoculation method, other factors such as soil microbes, temperature, relative humidity, growth medium, plant age and species, inoculum density and fungal species may be involved in a successful colonization of fungi in different plant tissues^28^.

Furtherly, PCR amplification of fungus-specified DNA band provides a new and sensitive method for fungal endophytes detection. For example, the FRR of *B. bassiana* was tested to be low by culturing plant tissues on fungal selective medium, but was tested to be 100% by PCR. Low endophytic fungal population density in plant tissues or biological inhibition from the plant tissues may contribute the failure of fungal growth on selective medium. The PCR amplification method could be reliably used in endophytic fungi study.

Previous studies revealed that some endophytic entomopathogens can promote plants growth as biofertilizers. Jaber et al.^16^ reported higher stem height, root length, fresh root weight, and stem weight than uninoculated plants 14 days after inoculating wheat seeds with *B. bassiana*. Russo et al.^30^ reported increased plant height, leaf number, plant height and the number of nodes on the first cob after spraying *B. bassiana* on maize leaves.

In our study, the two selected entomopathogenic fungi, *B. bassiana* and *M. anisopliae,* also significantly promoted the growth of maize in a plant hydroponic cultural system, and established a systematic colonization in different tissues in maize seedlings tissues for an expected long-term growth promotion.

In contrast, Moloinyane et al.^31^ did not find any significant differences in plant height, root and leaf number, fresh and dry weight between *B. bassiana* treated and untreated grapes even 4 weeks after soil drenching treatment. This is not surprising since the endophytic abilities of a particular fungal strain may be strongly associated with host plant species, plant species varieties^22^, nutritional conditions and environmental influences. Tall and Melying^32^ studied the effects of *B. bassiana* (GHA) seed treatment on maize growth. They found that *B. bassiana* can only be used as a growth promoter of maize only when nutrients were abundant, while no growth promotion effect was noticed in the absence of nutrients. So, mechanisms of plant responses to the fungal endophytism are far from clear and need further examination.

We studied entomopathogenic fungi, *B. bassiana* and *M. anisopliae* played their roles as maize growth promoters. However, it is unclear whether, rhizospheric or endophytic is the dominant mechanism? We tracked the population dynamics of *B. bassiana* and *M. anisopliae* in plant hydroponic solution and in plant tissues, hoping to shed light on the mechanism. In terms of CFUs, the populations of *B. bassiana* and *M. anisopliae* in the hydroponic solution rapidly decreased. After 1 week, a residual percentage of less than 10% of *M. anisopliae* and 1% of *B. bassiana* was detected, while both fungi were hardly detected on the 28th day in the maize culturing hydroponic solution. In a control study, both fungal conidia could maintain high viability after 1 week in the hydroponic culture system. Thus, the fungal endophytes, influenced by the conidia adhesion, host recognition and endogenous pathway^33–37^, was the main reason for the sharp decrease of fungal populations in the hydroponic culture system. Furthermore, the growth promotion function of the fungi was mainly due to the endophytic function rather than the rhizospheric function.

Normally, biological functions are density-dependent. The relationship between plant growth promotion and population density of endophytic fungi cannot be clearly clarified until the population of endophytic fungi in plant tissues could be quantitatively detected. The plant growth promotion mechanism still needs further exploration in the entomopathogenic fungi-plants interaction system. The entomopathogenic fungi exhibit great potential not only on both bio-control of pests but also on plant growth promotion, bringing us a new sight on ecological relationship among plants, insect pests, and entomopathogenic fungi.

## Methods

### Fungal strains and conidia suspension

The EPF strains *B. bassiana* Bb-13 and *M. anisopliae* Ma-73 were used in the study. These fungi are stored in the RCEF strain Bank, Provincial Key Laboratory of Microbial Control, Anhui Agricultural University.

The conidia suspension of *B. bassiana* and *M. anisopliae* strain were inoculated on PDA and SDAY medium respectively. After 14–18 days of cultivation, conidia were harvested by scraping conidia from the agar surface with a sterile inoculation shovel, and certain amount of conidia was put in sterilized 0.05% Tween-80 (v/v) and which was well mixed to make fungal conidia suspensions^38,39^. Conidial concentrations were determined using a hemacytometer and the final suspension was adjusted to 1 × 10^7^ conidia mL^−1^.

### Plants

Maize (*Z. mays,* Jingnuo No.1) seeds were purchased from Shouguang Yino Agricultural Science and Technology Co., LTD. The maize (100 seeds for one treatment) were seeded in breeding discs, each one with 32 holes (5.4 × 6 × 5 cm) containing sterilized culture substrates (Klasmann peat, German) (about 30 g of sterile soil is placed in per hole). One seed was placed per hole one seed. All maize seedlings are cultivated in a greenhouse for 14 days with automatically controlled environmental factors (25 ± 1 °C, 70 ± 5% RH and 16L: 8D regime).

### Fungal inoculation of maize seedlings

Ninety maize seedlings with even and well-good growth were randomly sampled for each treatment. The culture substrates on roots of each maize seedling were gently washed out with distilled water, avoiding any damage to the roots. The treated maize seedlings with even growth condition including aboveground and underground parts were transplanted to a maize hydroponic culturing system.

The maize hydroponic culturing system was constructed with an opaque plastic box (Working box specification: length × width × height of = 67 × 42 × 17.5 cm^3^) covered by an opaque plastic plate with 40 holes, in which 45L 1/2 half Hoagland nutrient solution (1/2) was added as hydroponic nutrient solution. A maize seedling was carefully transplanted in a culturing hole by fixing the plant stem with a sponge block^40^. Oxygen supply for the maize hydroponic culturing system was supported by a small ventilation pump (Risheng Group Co. LTD., CHN).

After two-day domestication for maize seedlings, 100 ml of conidia suspension with 1 × 10^7^ conidia mL^−1^ was added into each hydroponic solution. Eighty plants were used as the control, in which 100 ml of sterile 0.05% Tween-80 (v/ v) was added.

### Plant growth measurements

Maize seedlings were weekly sampled at random for each fungal treatment and the control. All plant growth indexes including plant height, root length, leaf length, leaf width, number of leaves, and fresh weight (the above-ground, underground and the total) were carefully measured^20^. The weight of above and below ground parts was separately weighed in an analytical balance (Yue Ping Scientific Instrument Co., LTD., CHN). All measurements were conducted by eight randomly sampled maize seedlings with two separate batches.

### Fungal population dynamic in hydroponic solution

Population density of *B. bassiana* and *M. anisopliae* in hydroponic solution were periodically monitored by the 7th day. One milliliter hydroponic solution was randomly sampled 5 times at a depth of 2 cm with a micropipette. The sampled hydroponic solutions were gradient diluted to 10, 100, 1000, 10,000 times with 0.05% Tween 80. After that, 100 uL of hydroponic solutions at 5 concentrations was sampled and evenly spread on a petri dish for fungal growth. Five repeats of BSM (*Beauveria* selective medium, quarter strength PDA containing 350 mgL^−1^ streptomycin sulfate, 50 mgL^−1^ tetracycline hydrochloride and 125 mgL^−1^ cycloheximide) petri dishes were used to assess the fungal population by terms of CFUs. The petri dishes were incubated at (25 ± 1) °C for fungal growth. The fungal colonies on the petri dishes were counted and identified. The optimal diluted concentration of the sampled hydroponic solution was the CFU number between 10 and 100 CFUs/plate^41^. The CFUs in the optimal range was used to value population dynamic in the hydroponic solutions.

### Determination of fungal colonization in maize tissues

#### Recovery of entomopathogenic fungi from maize tissues

The fungus was re-isolated by culturing tissues of the roots, stems and leaves of maize seedlings, inoculated with *B. bassiana* or *M. anisopliae as* rhizospheric fungus, on fungal selective medium to confirm fungal endophytic. Firstly, tissues of roots, stems and leaves were cut into pieces with 0.5 cm of length (roots and stems) or 0.5 cm^2^(leaves), and the small pieces of tissues were sterilized with 1% sodium hypochlorite for 5 min, then soaked in 75% ethanol for 1min^42^. Washed three times in sterilized distilled water, the tissue samples were inoculated on BSM medium and incubated at (25 ± 1) °C in the dark. Efficiency of the sterilization procedures of plant tissues was evaluated by inoculating 100 ul of the last rinse water on PDA medium to check any bacteria or fungi growth.

The BSM petri dishes were daily checked to find whether fungal colony appeared or not. Each growing fungal colony on BSM medium was inoculated on PDA or SDAY medium to identify the fungal species. Only those tissues with typical fungal colony of *B. bassiana* or *M. anisopliae* growth represented a successful fungal endogenesis in plants. Fungal recovery rates of *B. bassiana* or *M. anisopliae* in different tissues were calculated as the follows: Fungal recovery rate = [number of tissue pieces with *B. bassiana* or *M. anisopliae* colony/the total number of tissue pieces] × 100^43^.

#### DNA extraction of maize tissues

All maize tissues(100 mg) for DNA extraction were sampled and sterilized as the above procedure and then cut into small pieces with sterilized surgical scissors. Each tissue sample was transferred into a 1.5 ml centrifuge tube with steel balls added^39^. The tube was put into liquid nitrogen for 2 min frozen, and then the tissue sample was quickly ground for 150 s in an automatic grinding machine (Jingxin Technology Co., LTD, Shanghai). DNA was extracted from the sample tissue followed the steps of fungal genomic DNA extraction kit (Vokai Biotechnology Co., LTD., Beijing). The extracted DNA quality was examined with a micro nucleic acid protein detector (Wuzhou Oriental Technology Development Co., LTD., Beijing). The extracted DNA samples were stored at − 20 °C for PCR amplification.

#### PCR detection

All extracted DNA samples were evaluated by PCR amplification with specific primers of *B. bassiana* (F5'-TTCCGAACCCGGTTAAGAGAC-3', R5'-TTCCGAACCCATCATCCTGC-3')^44^ or *M. anisopliae* (5'GACTCTCTTAAGGTAGCCAAATGCC3', 5'AAACTCCCCACCTGACAATG-3')^45^. The PCR performed by the PCR Phire kit (Vazyme,Nanjing), and the PCR reaction system (20 µL) included 1.5 uL gDNA, 10 uL 2 × Tap master mix, 0.4 uL (10 uM) primer 1, 2, respectively and ddH_2_O added to 20 uL. The PCR amplification protocol for *B. bassiana* began at 95 °C for 3 min, followed by 35 cycles consisting of 15 s at 95 °C, 15 s at 55 °C, 25 s at 72 °C, followed by a 5 min extension at 72 °C, and for *M. anisopliae* was followed by 40 cycles consisting of 15 s at 95 °C, 15s at 65 °C, and 15 s at 72 °C. PCR products were kept at 4 °C, and detected by electrophoresis in 1% (wt/vol) agarose gels in TBE with ethidium bromide and visualized under UV (302 nm) light (BioRad, USA).

### Statistical analysis

All experimental data were analyzed using IBM SPSS Statistic (Version 20.0) for univariate ANOVA analysis, and Turkey HSD was used to test the significance of differences among treatments (P ≤ 0.05).

### Plant material collection and use permission

No permission is required for plant material as it was purchased from certified dealer of local area. The use of plants or plant materials in the present study complies with international, national and/or institutional guidelines.

### Ethics approval and consent to participate

The study has been conducted without violating any ethical codes of conduct.

## Conclusions

In summary, two entomopathogenic fungi, *B. bassiana* and *M. anisopliae,* played positive roles in promoting the growth of maize seedlings after hydroponic rhizosphere inoculation. The two fungi could establish systematic colonization in tissues of all maize organs through maize roots, and established within 1 week. Fungal population dynamics in hydroponic solution and fungal colonization in maize tissues suggested that the fungal endophytic function other than rhizospheric function contributes more to the observed plant growth promotion. Fungal endophytic behavior was found to be somewhat fungal species specific. The technique of PCR amplification of special DNA bands of the fungi is a more sensitive method than the fungal colony detection method by culturing plant tissues on fungal selective medium. The technique could be used to trace fungal colonization and spatial distribution in plant tissues with higher accuracy. Further study is still needed to elucidate the mechanisms of the responses of the plant and plant pests to the fungal endophytism (Supplementary Information).

## Supplementary Information

Supplementary Information.

## Data Availability

The datasets generated during the current study are available from the corresponding author on reasonable request.
