# Author Correction: Entomopathogenic fungi *Beauveria bassiana* and *Metarhizium anisopliae* play roles of maize (*Zea mays*) growth promoter

**DOI:** 10.1038/s41598-022-22870-1

**Published:** 2022-11-09

**Authors:** Yinmei Liu, Youkun Yang, Bin Wang

**Affiliations:** Provincial Key Laboratory of Microbial Control, Anhui Agricultural University, Hefei, 230036 China

Correction to: *Scientific Reports* 10.1038/s41598-022-19899-7, published online 20 September 2022

The original version of this Article contained errors.

In the legend of Figure 4, “*B. bassiana*” was incorrectly given as “*B. anisopliae*” and “*M. anisopliae*” was incorrectly given as “*M. anisoplia*”.

“PCR amplification of genomic DNA extracted from different plant parts of *B. anisopliae* and *M. anisoplia* treated *Zea mays* (**a**) Bb13; Lanes 1, 100 bp—1 kb DNA ladder; Lane 2–4, DNA from *Zea mays* root; Lane 5–7, DNA from *Zea mays* steam; Lane 8–10, DNA from *Zea mays* leaf. (**b**) Ma73; Lanes 10, 100—600 bp DNA ladder; Lane 1–3, DNA from *Zea mays* root; Lane 4–6, DNA from *Zea mays* steam; Lane 7–9, DNA from *Zea mays* leaf”.

now reads:

“PCR amplification of genomic DNA extracted from different plant parts of *B. bassiana* and *M. anisopliae* treated *Zea mays* (**a**) Bb13; Lanes 1, 100 bp—1 kb DNA ladder; Lane 2–4, DNA from *Zea mays* root; Lane 5–7, DNA from *Zea mays* steam; Lane 8–10, DNA from *Zea mays* leaf. (**b**) Ma73; Lanes 10, 100—600 bp DNA ladder; Lane 1–3, DNA from *Zea mays* root; Lane 4–6, DNA from *Zea mays* steam; Lane 7–9, DNA from *Zea mays* leaf”.

Additionally, in ‘Fungal endophytic, temporal and spatial distribution in maize tissues’, under the subheading ‘Isolation and identification of endophytic *B. bassiana* and *M. anisopliae* from different tissues of maize’, the citations to Fig. 3a and Fig. 3b were incorrectly given as ‘Fig. 3A’ and ‘Fig. 3B’.

“The fungal conidiogenous cell of *B. bassiana* formed a zigzag curve, and the conidia were spherical, transparent and smooth, consistent with the morphology of the pure strain *B. bassiana* Bb-13^21^ (Fig. 3A). The fungal colonies of *M. anisopliae* were white in the beginning and turned olive green after sporulation. The hyphae were transparent, separated and branched. Bottle-shaped conidiogenous cells formed a long string of conidia in basal continuity (Fig. 3B).”

now reads:

“The fungal conidiogenous cell of *B. bassiana* formed a zigzag curve, and the conidia were spherical, transparent and smooth, consistent with the morphology of the pure strain *B. bassiana* Bb-13^21^ (Fig. 3a). The fungal colonies of *M. anisopliae* were white in the beginning and turned olive green after sporulation. The hyphae were transparent, separated and branched. Bottle-shaped conidiogenous cells formed a long string of conidia in basal continuity (Fig. 3b).”

The original Article has been corrected.

