# Supplementary material for: Entomopathogenic fungi Beauveria bassiana and Metarhizium anisopliae play roles of maize (Zea mays) growth promoter
Source: Sci Rep. 2022 Sep 20;12:15706. doi: 10.1038/s41598-022-19899-7 (PMC9489797; doi:10.1038/s41598-022-19899-7)
Supplement: Supplementary file 1 — Supplementary Information. [file 41598_2022_19899_MOESM1_ESM.docx]

Scientific Reports

**Entomopathogenic Fungi *Beauveria bassiana* and *Metarhizium anisopliae* Play Roles of Maize (*Zea mays*) Growth Promoter**

**Yinmei Liu Author**^1，+^**, Youkun Yang Author**^1，+^**and Bin Wang Author** ^1,*^

^1^ Provincial Key Laboratory of Microbial Control, Anhui Agricultural University, Hefei 230036, China.

^*^corresponding. [bwang@ahau.edu.cn](mailto:bwang@ahau.edu.cn)

^+^these authors contributed equally to this work

| **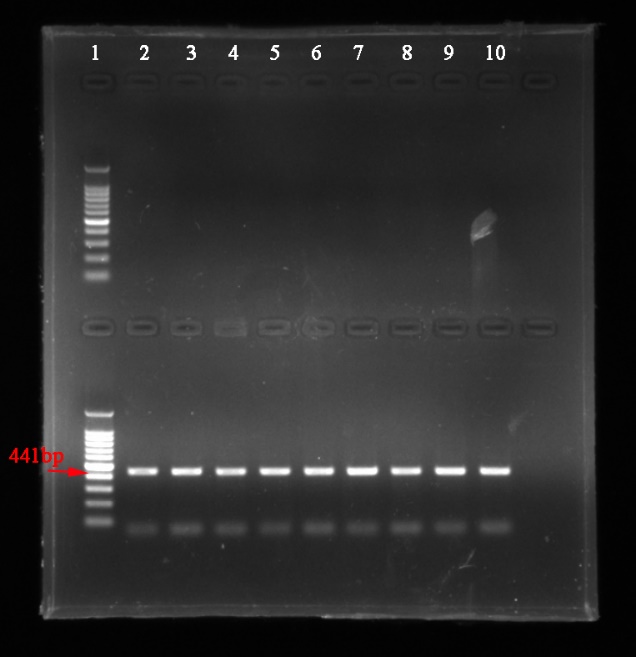**a | 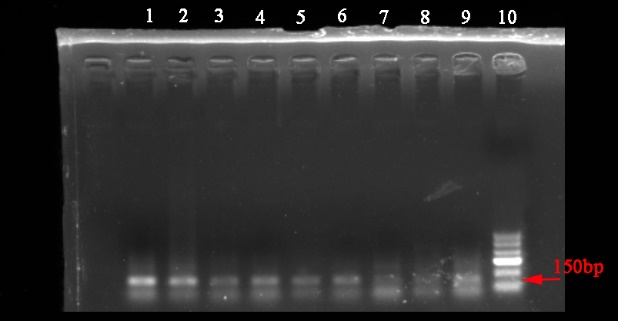b |
| --- | --- |

**Point 1：**We have reviewed the figures provided with the manuscript and must ask that the following editorial requests are addressed during revision:• We note that provided gel images look cropped **(Figure 4ab).**Are you able to provide images showing full-length membranes, with membrane edges visible? We ask that as full as possible length gels and blots with membrane edges visible are included in the Supplementary Information file. These images should be the original, unprocessed versions.

**Responds 1:** The following is the gel diagram of Figure 4ab.

**Fig.4**

**Point 2：**We notice that figures 3 A, B, C, D, E, and F have not been referenced in the main text of your manuscript file. If figures are not cited in the manuscript they will not appear in the HTML (online) version if your paper is accepted for publication. Therefore, it is essential they are mentioned at least once in the text and, we strongly recommend, in the order in which they are numbered.

**Responds 2:** Thank you for your suggestion. Annotations have been added in the corresponding position of the manuscript, as shown below.


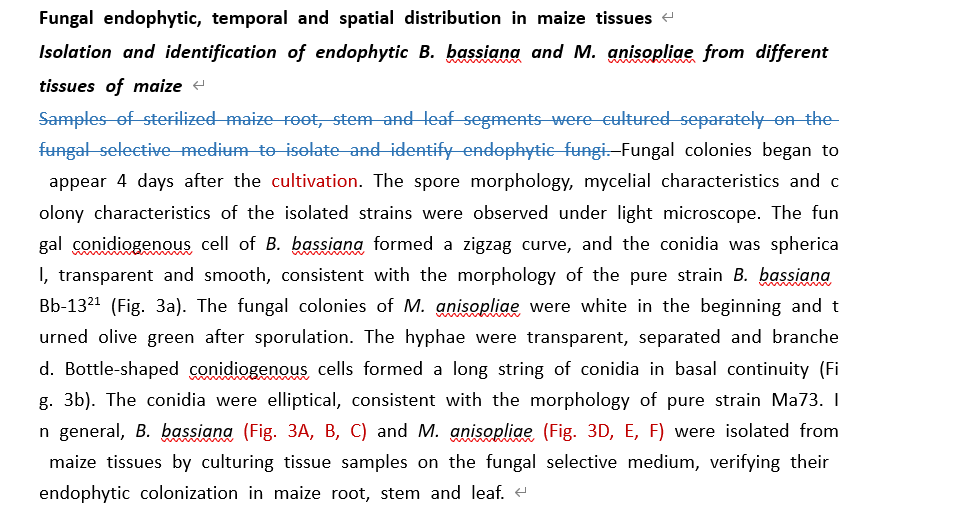


In addition, quality of two images submitted as Figure1,3 were improved to be 300DPI.

**Fig.1**


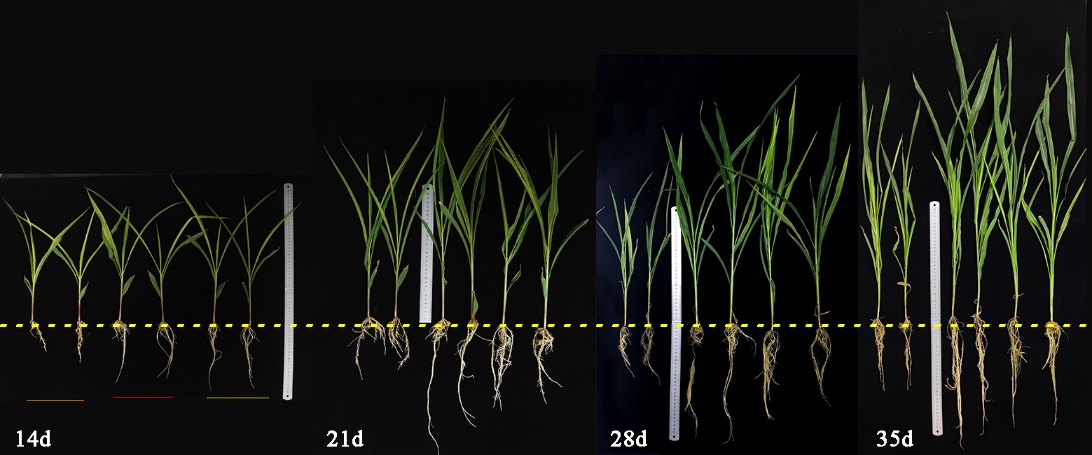


**
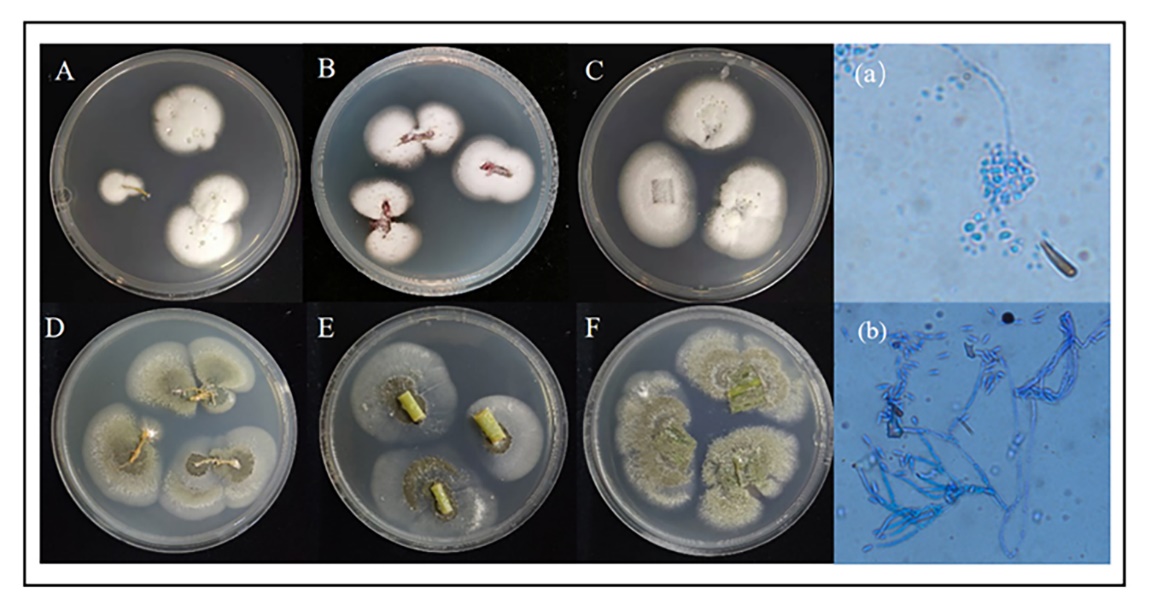
Fig.3**
